# Modulation of Prepulse Inhibition and Startle Reflex by Emotions: A Comparison between Young and Older Adults

**DOI:** 10.3389/fnagi.2016.00033

**Published:** 2016-02-23

**Authors:** Jolyanne Le Duc, Philippe Fournier, Sylvie Hébert

**Affiliations:** ^1^School of Speech Pathology and Audiology, Faculty of Medicine, Université de MontréalMontréal, QC, Canada; ^2^BRAMS, International Laboratory for Research on Brain, Music, and Sound, Université de MontréalMontréal, QC, Canada; ^3^CRIUGM, Centre de Recherche de l’Institut Universitaire de Gériatrie de MontréalMontréal, QC, Canada

**Keywords:** acoustic startle reflex, prepulse inhibition, aging, young, emotions, international affective picture system, eyeblink, positivity effect

## Abstract

This study examined whether or not the acoustic startle response and sensorimotor gating may be modulated by emotions differentially between young and older adults. Two groups of participants (mean age Young: 24 years old; Elderly: 63.6 years old) were presented with three types of auditory stimuli (Startle alone, High or Low frequency Prepulse) while viewing pleasant, neutral, or unpleasant images. Electromyographic activity of the eyeblink response was measured. Results show that older adults displayed diminished eyeblink responses whereas younger adults displayed enhanced eyeblink responses when viewing negative images. Sensorimotor gating also differed between young and older adults, with enhanced sensorimotor gating abilities while viewing positive pictures in older adults and diminished abilities while viewing negative pictures among younger adults. These results argue in favor of a differential emotional influence on the sensorimotor abilities of young and older adults, with a positivity bias among the latter.

## Introduction

The acoustic startle reflex is a cross-species, whole-body reflex in response to a loud and unexpected sound. Its basic circuitry is relatively simple (Cook et al., 1991; Kofler et al., 2001) and it is easily measured in humans by the magnitude and latency of the eyeblink, one component of the startle response. The presentation of a soft, non-startling sound (a “prepulse”) 30–500 ms before the startling sound reduces the startle reflex (Braff et al., 2001). Prepulse inhibition (PPI) reflects a fundamental protective function by reducing disruptive influences to the processing of prepulse signals, and is impaired in various cognitive disorders such as schizophrenia (Braff et al., 1978). It is widely acknowledged that the PPI paradigm provides an operational measure of pre-attentive sensorimotor gating. PPI itself can be modulated via descending projections from central brain structures such as auditory cortex and limbic circuitry (Li et al., 2009). This modulation determines how strongly the prepulse will inhibit the subsequent neural response from the facial motor nucleus responsible of the startle response (Swerdlow et al., 2001). The purpose of the present study was to characterize the interaction between emotion and aging on the startle reflex and its inhibition by a prepulse.

The general consensus among the many studies investigating the influence of emotions on the startle reflex is that it is reliably modulated by the affective valence of the emotions, i.e., pleasant vs. unpleasant (Vrana et al., 1988; Bradley et al., 1990, 1996b; Lang et al., 1990; Lang, 1995; Vanman et al., 1996; Hawk and Cook, 1997, 2000; Bradley and Lang, 2000; Roy et al., 2009), including fear (for a review, see Grillon and Baas, 2003). When exposed to pleasant, neutral or unpleasant stimuli (pictures or sounds), young adults show a typical pattern consisting of a stronger startle reflex during unpleasant than under neutral emotion, and a weaker one during pleasant than under neutral condition (Vrana et al., 1988; Bradley et al., 1990, 1993, 1996a,c; Feng et al., 2011). This pattern of responses is interpreted in terms of appetitive vs. aversive states, negative affective states engaging the defensive system that entails potentiation of the startle reflex, and positive affective states engaging the appetitive system that diminishes the startle reflex (Lang et al., 1990).

When considering the influence of age and emotion on the startle reflex, the appetitive/aversive model does not apply in a straightforward fashion. Accordingly, a recent study reported opposite eyeblink patterns for young and old adults: young adults displayed the typical pattern, that is, potentiated eyeblink when viewing unpleasant pictures whereas older adults displayed a reduced eyeblink when viewing the same pictures (Feng et al., 2011).

The possibility of a joint influence of emotions and age on PPI is important to consider since PPI is widely used to investigate pathological conditions such as schizophrenia—usually encountered in young people—and Alzheimer’s disease—found in the elderly (Braff and Geyer, 1990; Kumari et al., 2007; Wang et al., 2012). If sensorimotor gating were influenced by affective states or aging, these might become confounding variables in the analysis of PPI deficits. In the present study, young and older adults were asked to view pleasant, neutral or unpleasant pictures retrieved from the International Affective Picture System (IAPS; Bradley and Lang, 2007) paired with acoustic startle and prepulse sounds. Based on previous work (Feng et al., 2011), our expectation was that negative pictures would potentiate the startle reflex response in the young but not in the elderly. Given the paucity of available information regarding the influence of age and emotion on PPI (e.g., Hawk and Cook, 2000, who found no effect of emotions on PPI in young adults), our only expectation was that—if positive and negative emotions are processed differently in young and older adults–, PPI in older adults should be different for positive compared to neutral and negative images, and either the opposite pattern or no effect of emotions in young adults.

## Materials and Methods

### Participants

Two groups participated in the study: 26 Young (mean age of 24 years; range = 20–29, SD = 2.6) and 29 Elderly adults (mean age of 63.6 years; range = 56–69, SD = 3.6). An additional 7 young and 17 elderly adults were tested but were eliminated because they were considered non-responsive to the startle paradigm (see “Data Processing” Section below for details). Four young and one elderly participants were excluded on the basis of noisy EMGs or too many spontaneous blinks just before the startling stimuli. Participants were recruited through posted ads, word of mouth, as well as BRAMS’ and CRIUGM’s participant pools. The two groups were similar in gender (9 men in the Young and 10 in the Elderly group) and depressive symptomatology, but the younger participants were slightly more educated than the elderly (Table 1). Preliminary analyses with years of education as a covariate did not yield any significant effect of this factor or interaction with any other factors. They all self-reported good psychological and physical health and had normal hearing or only mild hearing loss as confirmed by audiometry performed in the laboratory with clinical equipment and procedure (hearing thresholds <45 dB HL at any frequency between 250 Hz and 4 kHz in at least one ear) and normal or corrected-to-normal vision. Participants with uncontrolled medical conditions (e.g., hypertension, diabetes), psychological, psychiatric or neurological conditions (depression, mood disorders, anxiety, bipolarity, etc.) and medication intake affecting nervous system were excluded. None of the participants was a smoker. The study was approved by the local ethics committees of the Université de Montréal and Institut Universitaire de Gériatrie de Montréal and was conducted with informed and written consent from all participants.

**Table 1:** **Socio-demographic characteristics (standard deviation, SD) of Young adults and Elderly**.

	Young (*N* = 26)	Elderly (*N* = 29)	*p*-value
Age in years (SD)	24.0 (2.6)	63.6 (3.6)	<0.001
Male/Female	9/17	10/19	n.s.
Education in years (SD)	16.5 (1.8)	14.8 (3.1)	0.02
BDI-II (SD)	5.3 (4.0)	4.6 (5.3)	n.s.
Right ear
PTA low frequencies	6.3 (4.7)	9.4 (7.2)	n.s.
PTA mid frequencies	5.7 (4.5)	13.9 (10.7)	0.001
PTA high frequencies	4.8 (5.0)	28.4 (13.5)	<0.001
Left ear
PTA low frequencies	3.9 (4.7)	9.1 (7.1)	0.002
PTA mid frequencies	4.1 (4.9)	11.6 (7.0)	<0.001
PTA high frequencies	5.0 (4.9)	28.1 (13.4)	<0.001

*Pure tone threshold average (PTA) in dB HL (SD) for low frequencies (250, 500, 750 Hz), mid frequencies (1000, 1500, 2000 Hz) and high frequencies (3000, 4000, 6000, 8000 Hz)*.

### Stimuli and Apparatus

#### Questionnaire

The Beck Depression Inventory II (BDI-II; Beck et al., 1996) was used to rule out participants suffering from severe depression (scores ≥ 30; Cook et al., 1991).

#### Startle and PPI Stimuli

**Figure 1:**
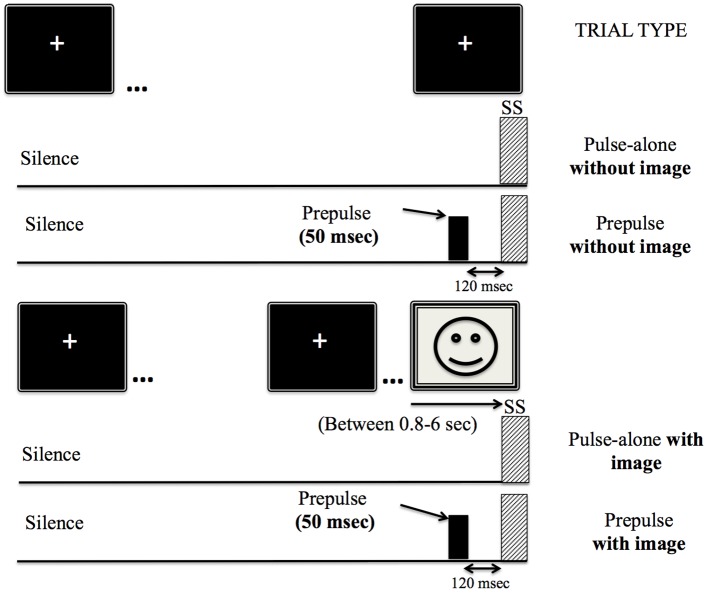
**Schematic representations of startle and prepulse trials**.

As previously reported (Fournier and Hébert, 2013), the auditory task consisted of three different stimuli (Figure 1): (i) Startle, a 50 ms broadband noise burst (20 Hz–20 kHz) set at 105 dB(A) SPL with near instantaneous rise-fall time (<1 ms); (ii) low frequency prepulses centered at 500 Hz (200–1200 Hz); and (iii) high-frequency prepulses centered at 4000 Hz (3.5–4.5 kHz). Two different prepulse sounds were used in order to maximize the probability of producing inhibition. The prepulse trials were 50 ms noise bursts presented at 65 dB(A) SPL with near instantaneous rise-fall time (<1 ms) that preceded a startle noise by 120 ms. The inter-stimulus interval (ISI) of 120 ms (SOA = 170 ms) was selected to maximize magnitude inhibition (Braff et al., 1978). The Intertrial-interval (ITI) time was randomly set at a value between 15 and 23 s in each block to decrease anticipation of the auditory stimulus (Patrick et al., 1993). All stimuli were presented in a silent background and created using Max/MSP Software program (Cycling 74, San Francisco, CA, USA).

#### Visual Stimuli

Originally 75 color photographs representing daily objects or situations that elicit emotions normally occurring in life were selected from the International Affective Picture System (Bradley and Lang, 2007). A pilot study was conducted involving 30 young (19–27 years old) and 27 older participants (55–80 years old) not included in this study but recruited using the same inclusion and exclusion criteria, in order to create two sets of stimuli comparable for the Young and the Elderly groups, as assessed with the Self-Assessment Manikin (SAM; Bradley et al., 1993; Bradley and Lang, 1994). Pictures were pleasant, neutral or unpleasant. To minimize the confounding effect of arousal on physiological responses, a subset of stimuli were chosen such that pictures from each emotional category did not differ in level of arousal between age groups. The final set thus included 66 pictures (22 pictures for each emotional category), with 59 pictures common to both groups and seven that differed across groups. While arousal ratings did not differ between age groups for any emotional category (*F*s < 1 for both Group and Group × Emotion), the corresponding valence, however, differed. Like several previous studies have reported (Grühn and Scheibe, 2008), older adults rated positive pictures as more positive than younger adults (with means of 7.93 and 7.13, respectively, *p* < 0.001), and negative pictures as more negative (with means of 1.79 and 2.19, respectively, *p* < 0.03). All pictures numbers are available upon request.

#### EMG Measurement

Eyeblink activity was measured using two 4 mm Ag/AgCl shielded recording electrodes positioned 1.5 cm apart on the orbicularis oculi muscle under the left eye and a ground electrode on the forehead, according to guidelines (Blumenthal et al., 2005). Signal acquisition was made using a IMac running the Acqknowledge 4.1 Software connected to a Biopac MP150 System (Biopac Systems, Inc., Santa Barbara, CA, USA) using the following settings: 1000× amplification, 90–500 Hz bandpass filter, RMS transformation, A/D conversion at 1 kHz. The stimulus presentation system was coupled to a Fireface sound card (RME, Haimhausen, Germany) hosted by a PC computer. Startle noise presentation was synchronized with eyeblink activity recording *via* a square-wave trigger signal to precisely determine the window of responses for magnitudes and latencies of the eyeblink (see “Data Processing” Section below).

### Procedure

Participants were instructed to sit quietly in a soundproof booth, refrain from moving and listen to the sounds presented binaurally *via* closed dynamic headphones DT 770 Pro/250 while watching a series of pictures projected on a dark screen. The test session began with a 2 min silent acclimatizing period followed by practice trials (four pulse-alone stimuli and one picture of each type). The task itself consisted of three equivalent blocks of trials of an approximately 8-min duration counterbalanced for pleasantness and arousal. Each block consisted of 21 auditory stimuli (seven startles, seven low-frequency and seven high frequency prepulses) and 21 pictures (seven pleasant, seven neutral and seven unpleasant). Six pictures of each type were pseudo-randomly combined with an auditory stimulus (e.g., two pleasant pictures with startle, two pleasant pictures with low-frequency prepulse, two pleasant pictures with high frequency prepulse per block). One auditory stimulus of each type was presented without any image for control purposes and one image of each type was presented without auditory stimulus to reduce predictability. Within each block, images were pseudo-randomly presented such that no more than two images with the same picture type (pleasant, neutral and unpleasant) were presented in succession. Participants viewed each image for 6 s, and the auditory stimulus arose randomly between 800 ms and 6 s after image onset. The first 800 ms were excluded since picture onset is known to cause PPI and reflect attention processes (Bradley et al., 1993). All stimulus types were calibrated before each testing session with an SE SoundPro DL 1/3 octave level meter (Quest Technologies, WI, USA) using an EC-9A artificial ear coupler (Quest Electronics, Oconomowoc, WI, USA) with appropriate rates, that is, impulse for startle noises/prepulse, using the A-weighting frequency curve. The total duration of the task was about 30 min.

### Data Processing and Statistical Analyses

For valence and arousal ratings in the pilot study, data above two SD from the group mean were replaced by the average value of the appropriate group (Young or Elderly) for each picture type (6.6%). Statistical analyses were run separately on valence and arousal. Data were entered into analysis of variances (ANOVAs) with Emotional condition (Unpleasant, Neutral, Pleasant) as within-subjects factors and Group (Young vs. Elderly) as the between-subject factor.

#### Startle and PPI Stimuli

All trials were visually inspected for excessive noise in the EMG signal and for any spontaneous blink occurring immediately before the startle stimuli. These trials were few (8.1% for Young adults and 6.8% for Elderly) and rejected from further analysis. For each participant, a baseline was defined from startle-alone trials only, by averaging the highest RMS amplitude value within a 200 ms window occurring 9–17 s before the startle noise onset, i.e., while no picture viewing. The peak-to-peak amplitude of each startle response 20–120 ms from pulse onset was extracted from the RMS data. Data for each trial type were averaged for each picture type for each participant. Any peak-to-peak amplitude value of any *trial* (i.e., prepulse or startle) <2 SD above the average baseline was considered a non-response and was assigned zero magnitude. In addition, only participants having a 100% response probability for startle trials were included in the study (final sample = 26 young and 29 elderly).

For raw EMG startle, data were entered in a mixed ANOVA with Emotional condition (Unpleasant, Neutral, Pleasant) and Group (Young vs. Elderly) as the between-subject factor. For Startle and PPI, *Z*-scores were also calculated to account for high levels of variability between groups in EMG responses (Patrick et al., 1993; Cuthbert et al., 1996; Feng et al., 2011). Startle and PPI *Z*-scores data > 2 SD from the group mean were replaced by the average value of the appropriate group (Young, Elderly) for each picture type (representing only 4.8% of all data). *Z*-scores were calculated for each participant using the formula *z* = *(x* − $\bar{x}$)/*s*, where *x* is the magnitude value of the trial, $\bar{x}$ is the mean response magnitude for all trials and *s* is the standard deviation of all trials.

Peak latency was obtained from the same time window but calculated from the raw EMG waveform following guidelines (Blumenthal et al., 2005). Raw latency data for startle and for PPI trials were entered in two separate mixed ANOVAs with Emotional Condition (Unpleasant, Neutral, Pleasant) as the within-subject factor and Group (Young vs. Elderly) as the between-subject factor. Latency facilitation was also calculated for each condition (Low- or High-Frequency prepulse) using the following formula: Latency facilitation = (pulse-alone latency) − (Low- or High-Frequency prepulse) latency. Latency facilitation data were entered in a mixed ANOVA with Emotional Condition (Unpleasant, Neutral, Pleasant) and Stimuli type (PPI High frequency, PPI Low frequency) as the within-subject factors and Group (Young vs. Elderly) as the between-subject factor. Peak rather than onset latencies were calculated because unlike onset latencies, peak latencies are not confounded by changes in reflex magnitude (Cadenhead et al., 1999).

All significant interactions and main effects were followed by appropriate ANOVA or *t*-tests. Bonferroni’s correction for multiple comparisons was used for *t*-tests when appropriate in order to keep the alpha level to 0.05 throughout all analyses, although original degrees of freedom are reported. Therefore, the *p* values reported in this article are corrected values. Effect sizes are reported for main effects with $\eta_{p}^{2}$ (Partial Eta squared). The no-image condition was compared between groups with independent sample *t*-tests.

## Results

### Startle

#### Raw EMG Data

**Figure 2:**
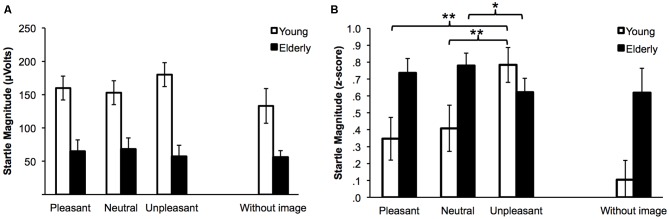
**Mean raw magnitude (A) and normalized data (B) of startle blink responses while viewing pleasant, neutral and unpleasant images for Young adults and Elderly.** The without-image condition is shown for comparison. Error bars are standard errors. *p < 0.05; **p < 0.01.

Figure 2A displays startle raw magnitude for the two groups and the three conditions. It is easily observable that Elderly had overall much smaller blink responses (*M* = 61 μV, SD = 81 μV) than Young adults (*M* = 157 μV, SD = 76 μV). This was supported by a main Group effect, *F*_(1,53)_ = 20.29, *p* < 0.001, $\eta_{p}^{2}$ = 0.277, and extends to startle trials not associated with pictures *t*_(30.4)_ = 3.51, *p* = 0.001, with means of 56 μV (SD = 37) and 133 μV (SD = 107), respectively.

#### *Z*-Scores

Figure 2B displays startle *Z*-scores for the two groups and the three emotional conditions. The main group effect was no longer significant, *F*_(1,58)_ = 2.21, *p* = 0.09, but the two-way interaction Emotion × Group was, *F*_(2,106)_ = 8.44, *p* < 0.001, $\eta_{p}^{2}$ = 0.137: as expected, Young adults displayed significantly greater startle reflex reactivity for unpleasant compared to neutral pictures, *t*_(25)_ = −2.98, *p* = 0.006, with means of 0.78 (SD = 0.5) and 0.41 (SD = 0.7), respectively, and to pleasant pictures, *t*_(25)_ = 3.4, *p* = 0.002, with a mean of 0.35 (SD = 0.6). Neutral and pleasant pictures did not differ (*t* < 1). For the Elderly, the only significant difference was between the neutral and unpleasant pictures, with lower startle reflex reactivity for unpleasant compared to neutral *t*_(29)_ = 2.32, *p* = 0.034 with means of 0.6 (SD = 0.4) and 0.78 (SD = 0.4), respectively. The difference between pleasant and unpleasant pictures failed to reach significance *t*_(29)_ = −1.4, *p* = 0.22, even though the startle reactivity to pleasant pictures was higher with a mean of 0.74 (SD = 0.5). Neutral and pleasant pictures did not differ (*t* < 1).

### Prepulse Inhibition

#### *Z*-Scores

**Figure 3:**
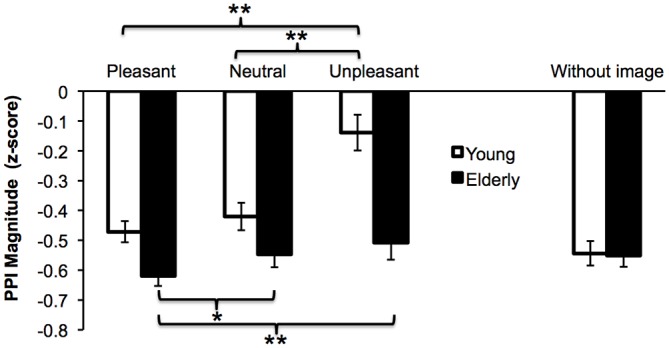
**Mean normalized PPI magnitude prepulses while viewing pleasant, neutral and unpleasant images for Younger adults and Elderly.** The without-image condition is shown for comparison. Error bars are standard errors. *p < 0.05; **p < 0.01.

Figure 3 displays PPI *Z*-scores for the two groups and the three emotional conditions. The two-way interaction was significant, *F*_(2,106)_ = 7.23, *p* = 0.001, $\eta_{p}^{2}$ = 0.120. Interestingly, a significant main effect of Group was observed, *F*_(1,53)_ = 16.33, *p* < 0.001, $\eta_{p}^{2}$ = 0.235, with overall more inhibition in the Elderly compared to the Young group. Following up on the two-way interaction, groups were analyzed separately and frequencies were merged. For the Young adults there was a significant main effect of Emotion, *F*_(2,50)_ = 14.68, *p* < 0.001, $\eta_{p}^{2}$ = 0.370, with negative images decreasing the inhibition significantly (*M* = −0.14, SD = 0.4) compared to positive images *t*_(25)_ = −4.58, *p* < 0.001 (*M* = −0.47, SD = 0.2) and neutral images *t*_(25)_ = 4.04, *p* < 0.001 (*M* = −0.42, SD = 0.3). For the Elderly group, there was a significant main effect of Emotion as well, *F*_(2,56)_ = 6.55, *p* = 0.003, $\eta_{p}^{2}$ = 0.190, with more inhibition while viewing pleasant (*M* = −0.6, SD = 0.1) than unpleasant *t*_(28)_ = −3.76, *p* = 0.001 (*M* = −0.5, SD = 0.2) and than neutral pictures *t*_(28)_ = −2.64, *p* = 0.013 (*M* = −0.5, SD = 0.2).

### Latency Data

Table 2 displays startle raw latency and latency facilitation data for the two groups in the three conditions and the without-image condition.

**Table 2:** **Latency of the startle reflex and latency facilitation of the prepulse inhibition values (in ms) for all emotional conditions for both groups**.

Measures	Groups	Conditions			
		Pleasant	Neutral	Unpleasant	No image
*Startle Latency in ms (SD)*	Elderly	*67 (7)	64 (8)	64 (8)	65 (9)
	Young	*59 (5)	57 (7)	57 (4)	61 (7)
*Latency facilitation in ms (SD)*	Elderly	11 (9)	5 (9)	7 (14)	6 (12)
	Young	3 (8)	3 (8)	3 (7)	6 (7)

*Startle latency is significantly longer for pleasant images than neural and unpleasant.

#### Startle Latency

The Elderly group displayed overall longer latencies compared to Young adults, as supported by a main Group effect, *F*_(1,53)_ = 30.8, *p* < 0.001, $\eta_{p}^{2}$ = 0.368 (Young: *M* = 58 ms, SD = 4, Elderly: *M* = 65 ms, SD = 6). A main effect of Emotion, *F*_(2,106)_ = 4.24, *p* = 0.017, $\eta_{p}^{2}$ = 0.074, revealed that irrespective of the group latency was greater for pleasant pictures (*M* = 63 ms, SD = 7 ms) than neutral (*M* = 61 ms, SD = 8 ms, *t*_(54)_ = 2.68, *p* = 0.015) and unpleasant pictures (*M* = 61 ms, SD = 7 ms, *t*_(54)_ = 2.51, *p* = 0.01). For the no image condition, there was a trend towards a slower reflex in the elderly but the difference was not significant *t*_(53)_ = −1.72, *p* = 0.092.

#### Latency Facilitation

On latency facilitation the three-way ANOVA revealed a significant main effect of Group, *F*_(1,52)_ = 6.67, *p* = 0.013, $\eta_{p}^{2}$ = 0.114, with the Elderly group displaying greater latency facilitation (that is, greater shortening of latency because of the prepulse) than Young adults (Elderly: *M* = 8 ms, SD = 6 ms; Young: *M* = 3 ms, SD = 5 ms). A significant two-way interaction between Frequency and Group also emerged, *F*_(1,52)_ = 4.40, *p* = 0.041, $\eta_{p}^{2}$ = 0.078. For low frequency only, the Elderly showed greater latency facilitation (*M* = 9 ms, SD = 9 ms) than Young adults (*M* = 2 ms, SD = 7 ms), *t*_(52)_ = −3.14, *p* = 0.003.

In order to examine the effect of sex as a confound variable, the analyses were repeated using sex as a covariate. There were no main effect of Sex or interaction with Sex for either startle reactivity or PPI (all *F*s < 1). The same results (i.e., no effect) was obtained when using Hearing thresholds (low, mid, or high PTAs; all *F*s ≤ 1).

## Discussion

Main findings are as follows: (1) emotions modulate sensorimotor gating abilities as assessed by the PPI paradigm; (2) the influence of emotions is different among younger compared to older adults; and (3) remarkably, such pre-attentional neurophysiological mechanisms are subject to a positivity bias among the elderly population. Indeed, the elderly displayed increased PPI when a positive state was induced whereas young adults displayed a reduced inhibition under negative state conditions. In both groups, the patterns of PPI responses were very different from the effect of emotion on startle reactivity. In agreement with a previous report (Feng et al., 2011), we found enhanced startle reactivity in young adults and reduced startle reactivity in the elderly when negative states were induced. These findings are not fully accounted for by the motivational priming hypothesis of Lang et al. (1990), that is, that the startle reflex is an aversive or defensive response. Rather, the response seems to be modulated by factors such as age.

The different patterns of response between the PPI and startle reactivity paradigms in both groups argue in favor of two different underlying neurophysiological processes as previously suggested. Hawk and Cook (2000) using a similar paradigm in undergraduate students found increased startle reactivity while participants were viewing negative pictures but without effect of emotion on PPI. One possible explanation for the divergence between their PPI results (the only comparable data available) and ours might lie in the stringent criteria used in the present study since we enforced explicit exclusion criteria known to influence the startle reflex such as smoking, hearing thresholds and depression, whereas Hawk and Cook (2000) did not specify any exclusion criteria based on health or lifestyle. More importantly, only participants having a 100% response probability for startle trials were included in our analyses, i.e., only those with a startle reflex on every non-rejected startle trials (<2 SD above baseline). This objective criterion was used to maximize the probability of finding an effect of emotion on startle reactivity (which we did, similarly to Feng et al., 2011) and PPI, both paradigms being investigated—for the first time—in all participants in the present study. Since groups differed in startle reactivity (older adults had smaller blinks than younger ones), this criterion ensured that our study was adequately powered with similar numbers of trials to detect differences between groups. Having examined both types of responses in the same individuals, we are therefore confident in the robustness of our findings. At the same time, the necessity of using such a strict criterion highlights methodological limitations of the startle and PPI techniques to assess emotional influence on sensorimotor gating process. Auditory evoked potentials could be used in follow-up studies looking at the interaction of emotion and aging on sensory gating process to avoid these limitations (Broyd et al., 2013; Smith et al., 2013).

Both sensorimotor gating and emotional processing seem to be well preserved in aging, as shown by similar levels of PPI in the no-image condition in young and older adults, and a strong effect of emotion regulation by the prepulse. Older adults have a bias towards positive emotional stimuli and decreased processing of negative emotional stimuli (the positivity effect, Mather and Carstensen, 2005), they remember more positive than negative information than young people (Charles et al., 2003) and display reduced late positive potential (LPP) amplitude for negative stimuli (Mathieu et al., 2014). When free to look at positive, neutral, or negative pictures, a higher proportion of pictures remembered by older adults consisted of positive than negative information compared with the younger adults. Older adults also display more fixations and of longer duration for positive compared to negative or neutral pictures, a pattern different from younger adults (Sasse et al., 2014). A recent meta-analysis including 100 empirical studies on the positivity effect showed that the effect is robust across a wide range of tasks and stimuli (Reed et al., 2014). Here, we add new data showing that this positivity effect can be shown at pre-attentive stages. A few brain imaging studies have also shown that older adults display greater amygdala activity for positive vs. negative pictures (Mather et al., 2004; Mather and Carstensen, 2005). Since both startle reflexes and PPI can be modulated by input from the amygdala (Koch, 1999), it is likely that the amygdala is one important relay for the effect described here, and that this effect is more easily observable through the simpler neural circuit of the startle reflex. Future studies should look at the contribution of this structure on the processing of emotions on both startle and PPI. A methodological implication of these findings is that since the emotional status can have a significant influence on physiological reactivity, studies looking at the differential effects of age on the acoustic startle and PPI should take into account the emotional state of their participants.

In conclusion, although older adults show a reduced and delayed acoustic startle response compared to young adults (consistent with previous studies, e.g., Kofler et al., 2001; Ellwanger et al., 2003), their responses are strongly modulated by emotions. Older adults display diminished startle reactivity while viewing unpleasant compared to neutral and pleasant images, which is the reverse pattern from younger adults who display a potentiated reflex while viewing unpleasant images compared to neutral and pleasant images. In addition, older adults display stronger sensorimotor gating while viewing positive compared to neutral or unpleasant images, a pattern different from the younger adults. These results argue in favor of a differential emotional influence on the sensorimotor abilities among young and older adults, with a positivity bias among the latter.

## Author Contributions

All authors listed, have made substantial, direct and intellectual contribution to the work, and approved it for publication.

## Conflict of Interest Statement

The authors declare that the research was conducted in the absence of any commercial or financial relationships that could be construed as a potential conflict of interest.
